# DisCons: a novel tool to quantify and classify evolutionary conservation of intrinsic protein disorder

**DOI:** 10.1186/s12859-015-0592-2

**Published:** 2015-05-13

**Authors:** Mihaly Varadi, Mainak Guharoy, Fruzsina Zsolyomi, Peter Tompa

**Affiliations:** VIB Structural Biology Research Center (SBRC), Brussels, Belgium; Vrije Universiteit Brussel, Brussels, Belgium; Institute of Enzymology, Research Centre for Natural Sciences, Hungarian Academy of Sciences, Budapest, Hungary

**Keywords:** Intrinsic protein disorder, Large-scale sequence analysis, Molecular recognition features (MoRFs), Short linear motifs (SLiMs)

## Abstract

**Background:**

Analyzing the amino acid sequence of an intrinsically disordered protein (IDP) in an evolutionary context can yield novel insights on the functional role of disordered regions and sequence element(s). However, in the case of many IDPs, the lack of evolutionary conservation of the primary sequence can hamper the study of functionality, because the conservation of their disorder profile and ensuing function(s) may not appear in a traditional analysis of the evolutionary history of the protein.

**Results:**

Here we present DisCons (*Dis*order *Cons*ervation), a novel pipelined tool that combines the quantification of sequence- and disorder conservation to classify disordered residue positions. According to this scheme, the most interesting categories (for functional purposes) are constrained disordered residues and flexible disordered residues. The former residues show conservation of both the sequence and the property of disorder and are associated mainly with specific binding functionalities (e.g., short, linear motifs, SLiMs), whereas the latter class correspond to segments where disorder as a feature is important for function as opposed to the identity of the underlying sequence (e.g., entropic chains and linkers). DisCons therefore helps with elucidating the function(s) arising from the disordered state by analyzing individual proteins as well as large-scale proteomics datasets.

**Conclusions:**

DisCons is an openly accessible sequence analysis tool that identifies and highlights structurally disordered segments of proteins where the conformational flexibility is conserved across homologs, and therefore potentially functional. The tool is freely available both as a web application and as stand-alone source code hosted at http://pedb.vib.be/discons.

## Background

Intrinsically disordered proteins (IDPs) and intrinsically disordered regions (IDRs) within structured proteins are defined by the lack of a stable tertiary structure and a corresponding high degree of flexibility under physiological conditions [1]. The importance of conformational flexibility is reflected in the observation that IDPs and proteins with IDRs are often involved in essential cellular processes, such as cell-cycle regulation, transcription, and translation [2-4]. Additionally, they often play major roles in pathologies associated with aggregation and misfolding [5,6], making them attractive potential drug targets [7]. Genes encoding such amino acid sequences are under reduced selective pressure, which is manifest in a higher sequence diversity compared to genes of structured proteins/domains [8]. Whereas the functionality of a protein segment is often approached by investigating the evolutionary history of its primary sequence [9], this is often difficult to achieve with IDP/IDR sequences, due to their generally high sequence diversity [10].

On the other hand, combining the information derived from analyzing the conservation of both sequence and disorder can be much more useful, and this idea has recently been suggested to partition disordered residue positions into three separate groups of potentially different functional attributes: i) ‘constrained’, if both features (amino acid sequence and the property of disorder) are conserved; ii) ‘flexible’, if only disorder is conserved; and finally, iii) ‘non-conserved’ positions where disorder is not conserved. These specific evolutionary behaviors have been shown to correlate with distinct disorder-related functional categories [11]. In general, segments of constrained disorder are often associated with protein binding and molecular recognition, whereas flexible disorder is prevalent in linker segments acting as entropic chains. Non-conserved disorder has not been associated with specific protein function so far.

Here, we present DisCons, a novel web application and downloadable, stand-alone source code that offers a description of the conservation of both the amino acid sequence and of the feature of structural disorder, and performs the classification of disordered positions into these three categories. Thus, DisCons provides an additional (integrative) layer of information that together with other sequence-based tools, such as the PAML software package [12], MoRFpred [13] and Anchor [14], should facilitate the effective identification of functionally important disordered regions in proteins.

## Implementation

Both the web application and the downloadable version of DisCons hosted at the website are freely available without registration. The DisCons website is divided into four functional sections that are accessible both through the menu and via the options shown on the welcome page. These sections correspond to the three running mode interfaces: ‘quick’, ‘advanced’ and ‘from alignment’. The fourth functional section is ‘help’, which offers the complete documentation of the server and source code, in addition to a user guide.

The ‘quick’ running mode requires a single protein sequence (in FASTA format) as input, or alternately, a UniProt [15] accession ID. In this mode, the default parameters are used through all the calculation steps of the DisCons workflow. Although this calculation is the easiest to set up, experienced users might prefer to use the ‘advanced’ tool enabling a better understanding of the results leading to more fine-tuned functional interpretations.

The ‘advanced’ mode also accepts a single protein sequence or UniProt ID in a manner similar to the ‘quick’ calculation, but in this mode users can manually set all the parameters of the underlying calculation, allowing for a detailed optimization of the protocol pipeline, and a better overall command of the final results.

Finally, the ‘from alignment’ mode is best suited if the user already has a custom made, reliable multiple sequence alignment that can be used for the calculations. The main advantage of this mode is speed, since the need for running a BLAST search and constructing the multiple sequence alignment with MAFFT (which are the most time-consuming of all the steps) is circumvented. Therefore, this mode is significantly faster than the others which start from a single sequence (although even in ‘quick’ and ‘advanced’ modes, the approximate time for generating the results is 34 seconds for a ~2400 residue long protein). By default, the stand-alone source code is also running ‘from alignment’; however if the necessary dependencies, namely BLAST+ [16] and MAFFT [17] are available locally in the user’s computer, the full pipeline can be utilized in a straightforward manner.

**Figure 1:**
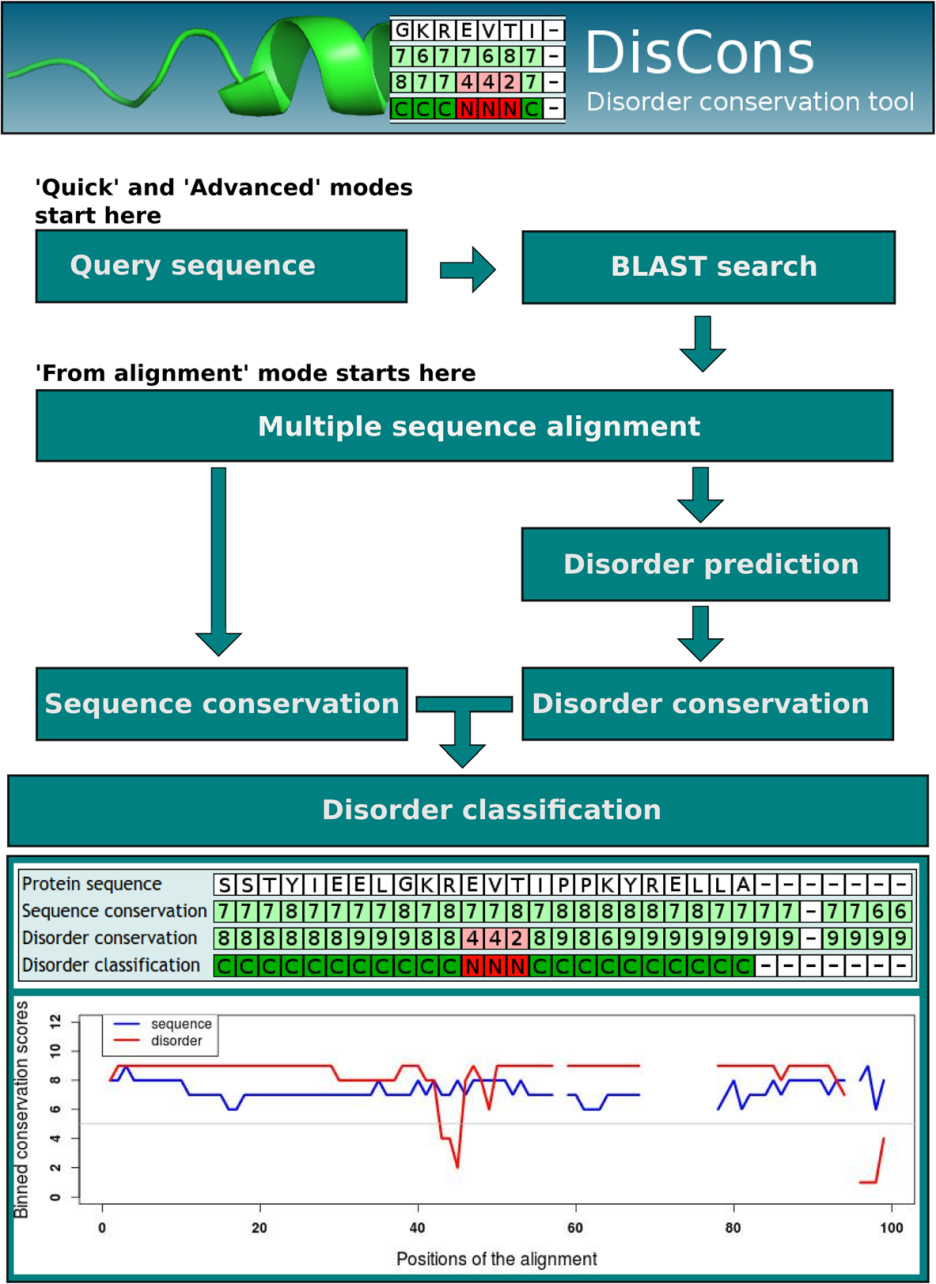
Discons Work Flow. The schematic work flow of the DisCons sequence analysis pipeline is displayed along with a sample output table, showing the DisCons profile of human calpastatin domain I [Swiss-Prot:P20810]. The procedure starts either from a query sequence, followed by a BLASTP or PSI-BLAST search and the aligning of the retrieved sequences, or from a user-provided multiple sequence alignment. Next, position-specific sequence- and disorder conservation scores are calculated and finally these pairwise scores are combined to create the (aligned) DisCons profile. The result of one such classification is displayed at the bottom of the figure.

Depending on the running mode, the workflow of the calculations has a different starting point (Figure 1). In ‘quick’ and ‘advanced’ modes, the procedure starts with a BLASTP or PSI-BLAST search [16] to collect sequences similar to the query sequence. In ‘advanced’ mode, the search dataset (Swiss-Prot [15] (used by default) or PDB [18]) and the BLAST threshold values can be specified. Next, a multiple sequence alignment (MSA) is created from the set of identified homologous sequences using MAFFT [19]. This is the most crucial part of the procedure, since aligning disordered regions is non-trivial due to the potential diversity of related sequences. Because an incorrect alignment will compromise the subsequent calculations, it is advised either to use the ‘advanced’ mode to fine-tune the alignment procedure or to use a reliable, user-defined multiple alignment in the ‘from alignment’ mode.

In the next step (which is the starting point when running in the ‘from alignment’ mode), the MSA is used to construct an aligned disorder profile by running IUPred (default) [20], VSL2 [21], ESpritz [22] or FoldIndex [23] on each of the aligned sequences. The disorder scores are first transformed to a binary scale (1 = disordered, 0 = ordered; residues with a disorder score of 0.5 or greater are considered as disordered) for each sequence. Details about the transformation procedure for the three different disorder predictors are given in the help section of the website. Next the fraction of disordered residues at every position in the MSA across the different sequences is calculated, thereby effectively quantifying the position-wise conservation of disorder.

Next, sequence conservation scores for each position in the alignment are calculated using the algorithm developed by Capra et al. [9]. In ‘advanced’ and ‘from alignment’ modes, a number of parameters such as the algorithm of choice or the background distribution can be specified to make the calculation more robust.

Finally, positions in the MSA are scored by both their sequence- and disorder conservation, ranging from 0 (very diverse) to 9 (highly conserved) (Figure 1). Based on these pairs of scores, each position will fall into one of four distinct categories. As suggested by Bellay et al. [11], positions with a higher degree of disorder can be ‘constrained’ (‘C’), if both the sequence and disorder conservation scores are 5 or greater; ‘flexible’ (‘F’), if the sequence conservation is lower than 5 but the disorder conservation is 5 or greater; or ‘non-conserved’ (‘N’), if the disorder conservation is lower than 5, but higher than 0. Positions with a disorder conservation score of 0 are completely lacking disorder and therefore are considered as ‘structured’ (‘S’). Thus, regions of constrained disorder show strong conservation both at the amino acid level, and also of the disorder feature, while flexible disordered regions are variable in terms of amino acid sequence, but retain a significant level of disorder in evolution. Lastly, non-conserved disordered regions lack disorder as a conserved feature, and are generally thought not to be associated with functions [11].

On the results page, the position-specific conservation profile is provided on the output screen, and the fractions of residues falling into each of these distinct categories are also displayed in a tabular format at the bottom of the page, effectively quantifying the conservation of disorder in the query sequence (a part of such an output is shown in Figure 1). The sequences of consecutive ‘constrained’ disordered regions are also recorded and are available for download in FASTA format along with the profiles and fractions in text format using the links that are provided. Such segments of consecutive stretches of constrained disorder are most likely to correspond to functionally important IDRs such as linear motifs or MoRFs, as we describe below.

## Results and discussion

### Bench-marking on molecular recognition features

We evaluated the performance of DisCons on a set of molecular recognition features, or MoRFs, that are short peptide segments mostly found within longer disordered regions (LDRs) and involved in the binding to protein partners via disorder-to-order transition [13]. MoRFs have been implicated in functions involving regulation and signaling, among other cellular processes. These recognition features are enriched in disordered residues, however, they may also have some residual (transient) structure, and their sequences are relatively more conserved than their flanking disordered regions [24,25].

**Figure 2:**
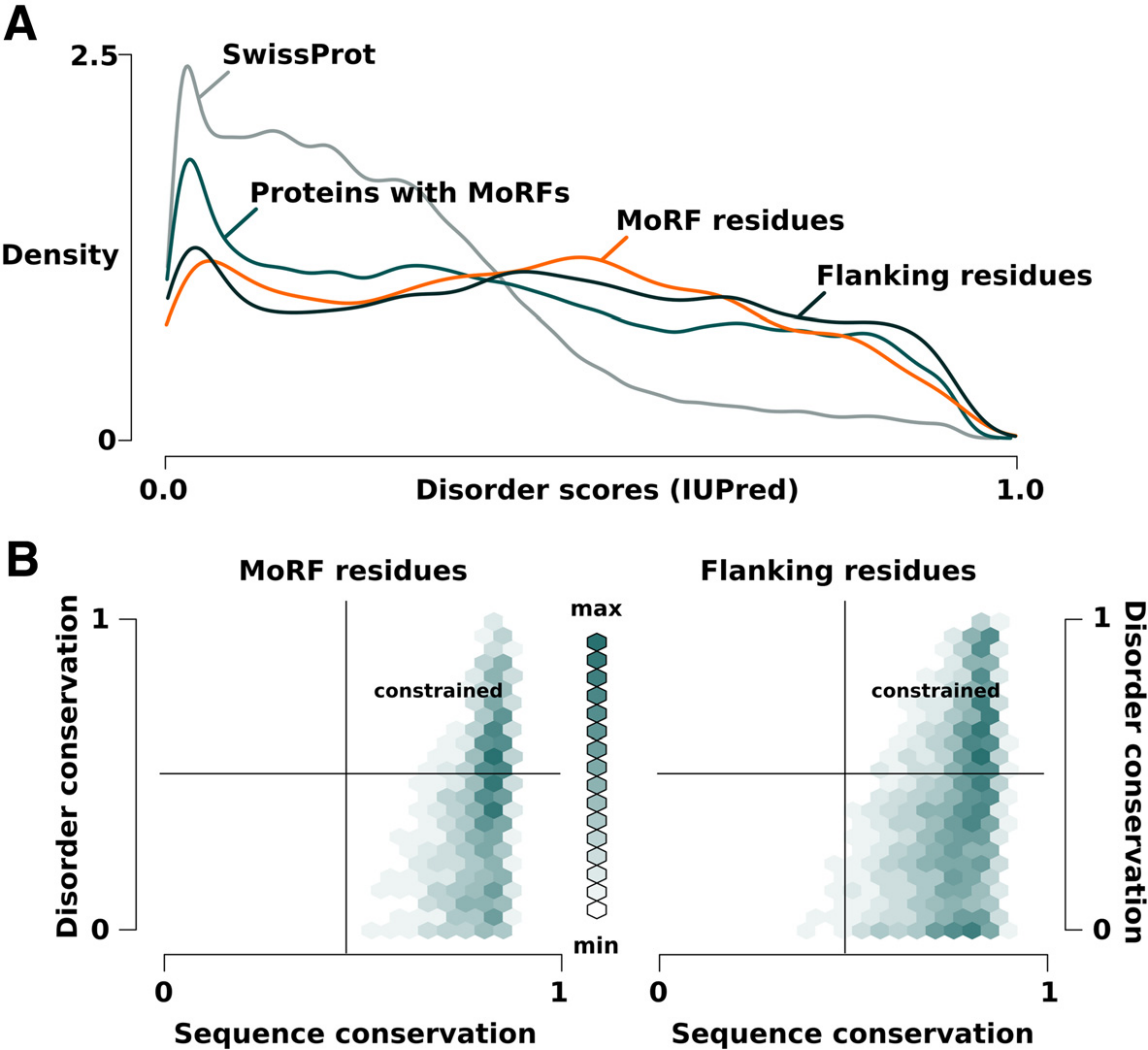
Analyzing MoRFs with DisCons. MoRFs are known to be enriched in disordered residues. Panel **A** shows normalized density distributions of MoRF regions, MoRF-flanking segments, full-length sequences of the MoRF-containing proteins and all proteins from Swiss-Prot. In these distributions, the area under each curve adds up to one. MoRF residues (orange) are predicted to be more disordered on average than the proteins they are found in, and especially more than the proteins of UniProt/SwissProt. The flanking regions (dark green) of the MoRFs also have significantly higher disorder content, compared to the full-length proteins they are found in. Panel **B** shows the combined disorder- and sequence conservation scores, which range from 0 (not conserved) to 1 (conserved at all positions in the multiple sequence alignment). By comparing these residue-specific score pairs, DisCons further supports the idea that the sequences of MoRFs (left) are more conserved than that of their flanking regions (right); however, even in the flanking regions intrinsic disorder as a feature is highly conserved, indicating that these segment are required to be flexible in order for the protein segment to function.

To estimate the efficiency of the DisCons protocol in distinguishing between such functionally important disordered segments on a large scale, we retrieved three MoRF datasets from MoRFpred that are available at their website [13] and combined them into a single benchmarking dataset. The three datasets were the ‘test dataset’ containing MoRFs deposited in the Protein Data Bank (PDB) before 2008; the ‘experimental dataset’ with MoRFs identified between 2008 and 2012; and the ‘test 2012’ dataset with MoRFs from 2012. The combined dataset contained 469 MoRF instances. After applying a sequence redundancy filter on the full length sequences using CD-hit [26], 416 unique sequences remained. MoRF sequences were extracted from the full length protein sequences along with up to 30 residue long flanking segments on both sides. Disorder propensity scores were then calculated for the extracted MoRFs, the flanking residues, the full length proteins, and the complete UniProt/SwissProt database, using IUPred. Figure 2A displays the distribution of disorder scores, comparing these four datasets, demonstrating that MoRF residues (median: 0.43) and MoRF-flanking regions (median: 0.45) are more disordered than MoRF-containing proteins (median: 0.37). The difference is even more pronounced when compared to proteins from the complete UniProt/SwissProt database (median: 0.22). Since the distributions did not follow Gaussian or even symmetric distribution, we chose the non-parametric Kolmogorov-Smirnov (KS) test, which only assumes that the compared variables are continuous. The distributions of MoRFs and flanking residues are significantly different from both their proteins and the UniProt/SwissProt dataset, according to KS tests with p-values less than the precision limit of R (p-value < 2.2e-16), indicating very strong significance.

After performing PSI-BLAST [16] searches against each MoRF and their flanking regions, the conservation of the aligned positions were quantified in terms of sequence- and disorder conservation according to the protocol explained in the ‘implementation’ section. The binned conservation score pairs are displayed on Figure 2B, comparing MoRFs (left) and flanking residues (right). In order to statistically compare the conservation scores, we used the Welch *t*-test, since this test only assumes Gaussian distribution of the variables, and does not require equal variances. The conservation of disorder in MoRF (mean = 0.39) and flanking (mean = 0.4) residues is similar (Welch *t*-test p-value = 0.056), while the underlying amino acid sequence is significantly more conserved in the case of MoRF residues (mean = 0.79 as opposed to 0.74 of the flanking segments, Welch *t*-test p-value < 2.2e-16). Therefore, the comparison of MoRFs and MoRF-flanking sequences shows a trend that is in agreement with the literature [24,25], namely that while both sequence and disorder are rather conserved in the MoRFs, their neighboring protein segments are less conserved sequence-wise.

### Bench-marking on short linear motifs

**Figure 3:**
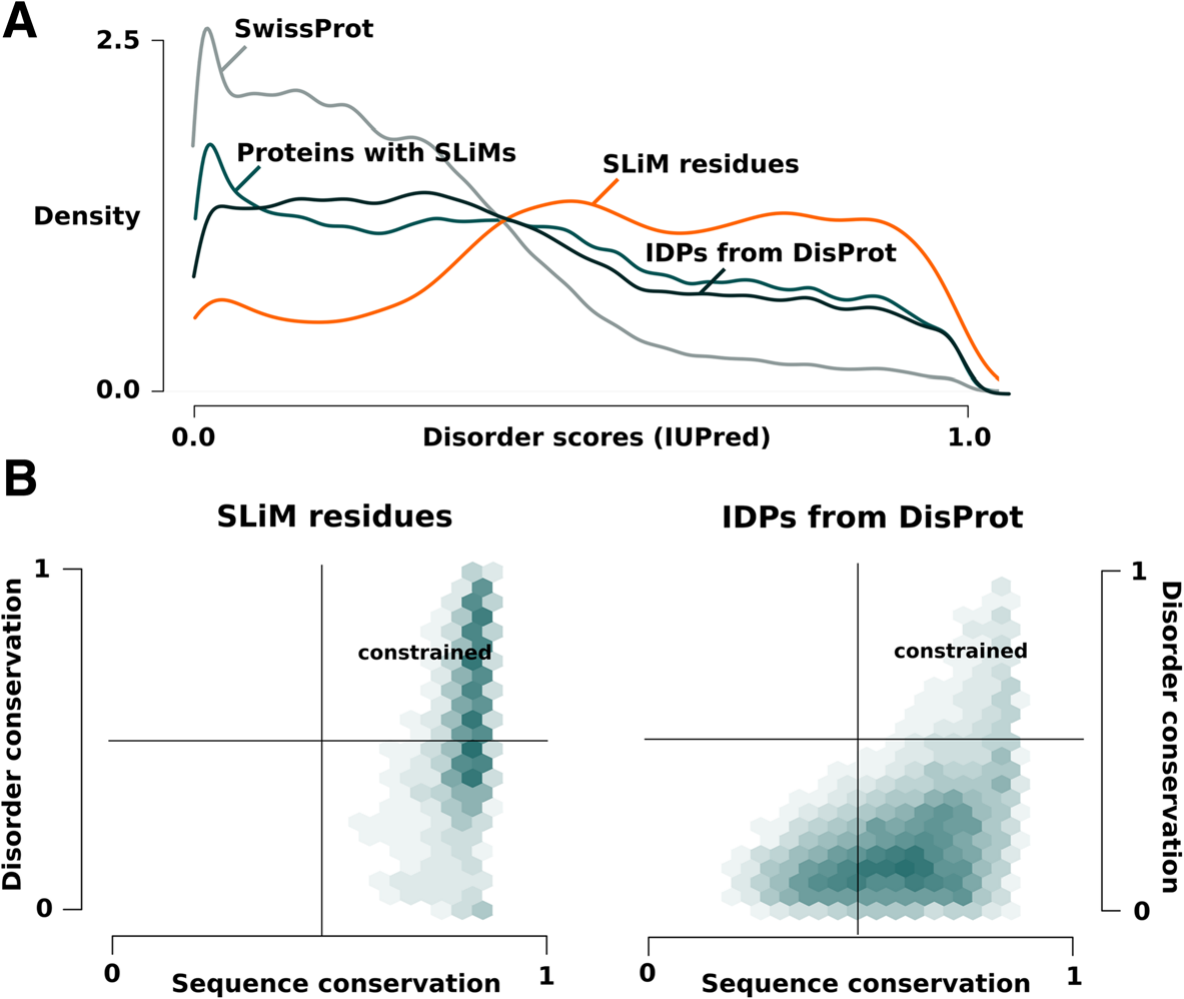
Analyzing SLiMs with DisCons. Short linear motifs (SLiMs) are peptide motifs with characteristic sequence patterns and are generally enriched in disorder. As displayed on panel **A** with the help of normalized density curves, SLiMs are significantly more disordered than the full length proteins they are found in or compared to the proteins found in DisProt. Not only are SLiMs more disordered, but this flexibility is highly conserved across homologs as well (panel **B**, left). When compared to the IDPs of DisProt (panel **B**, right) the difference in conservation is striking. As in Figure 2B, disorder- and sequence conservation scores range from 0 (not conserved) to 1 (conserved at all positions in the multiple sequence alignment).

Following the analysis of the MoRF dataset, we applied the DisCons procedure on all the 1590 known instances of short linear motifs (SLiMs) of the ELM database [27]. Generally, these motifs are enriched in disorder, and their sequences show higher than average conservation on the amino acid level [28]. We compared these motifs to all the available IDP sequences retrieved from DisProt [29] (Figure 3). The average disorder content of SLiMs is significantly higher than that of the full length IDPs (KS test p-value < 2.2e-16), and in fact even more so than in the MoRF dataset (KS test p-value < 2.2e-16) (Figure 3A). As expected and demonstrated in Figure 3B, both the sequence and disorder conservation scores of the SLiM sites are significantly higher than that of the full length IDPs (both Welch t-tests with p-values < 2.2e-16), with 52% of all the SLiM residues being of constrained disorder compared to only 12% in the full length IDPs, indicating the importance of structural disorder in SLiMs. In comparison to MoRFs, where 36% of the residues are of constrained disorder, structural flexibility seems to play a larger role in SLiMs, and indeed, MoRFs are known to often have some residual pre-formed structural elements, while SLiMs are more disordered overall. Additionally, the majority of SLiMs are localized on consecutive segments of constrained disorder. Concretely, 88% of the SLiMs were found within consecutive segments of 5 or more constrained residues, 74.4% in segments that are at least 10 residues long, and 55.5% in segments of at least 20 constrained residues in length. Since SLiM segments often form ligand/protein binding sites, their high sequence conservation is necessitated by the formation of interface contacts with partner proteins.

### Case studies of DisCons uncovering constrained and flexible disorder

**Figure 4:**
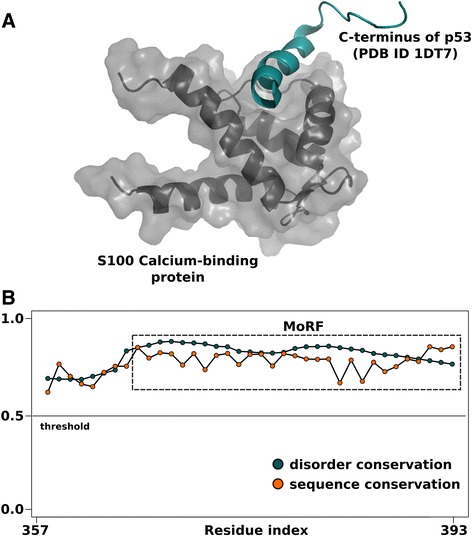
Example of a constrained disordered MoRF. The intrinsically disordered C-terminus of p53 adopts a helical fold upon partner binding (panel **A**). This is the only segment of the disordered region that appears in this crystal structure. Upon quantifying the conservation of sequence and of disorder in the C-terminus, the full length MoRF segment is classified as ‘constrained’ disordered, where both features are highly conserved (panel **B**).

Finally, we provide two case studies using two different protein segments; one exemplifying “constrained” disorder and the other being an example of the “flexible” disorder class. Figure 4A shows a MoRF region of constrained disorderfound in the C-terminal negative regulatory domain of the p53 protein (colored cyan) bound to the S100 Calcium-binding protein [PDB:1DT7], along with the DisCons profile of the C-terminal part of the p53 sequence (Figure4B). All the residues forming the MoRF are constrained based on the conservation profile of sequence- and of structural disorder, and, not surprisingly, these are the only residues of the disordered segment that appear in the crystal structure. The interaction with S100 restricts access to phosphorylation and acetylation sites on p53 that are important for transcription activation [30]. Thus, this region that is important for mediating a critical interaction is clearly identified by our protocol as a conserved disordered segment.

**Figure 5:**
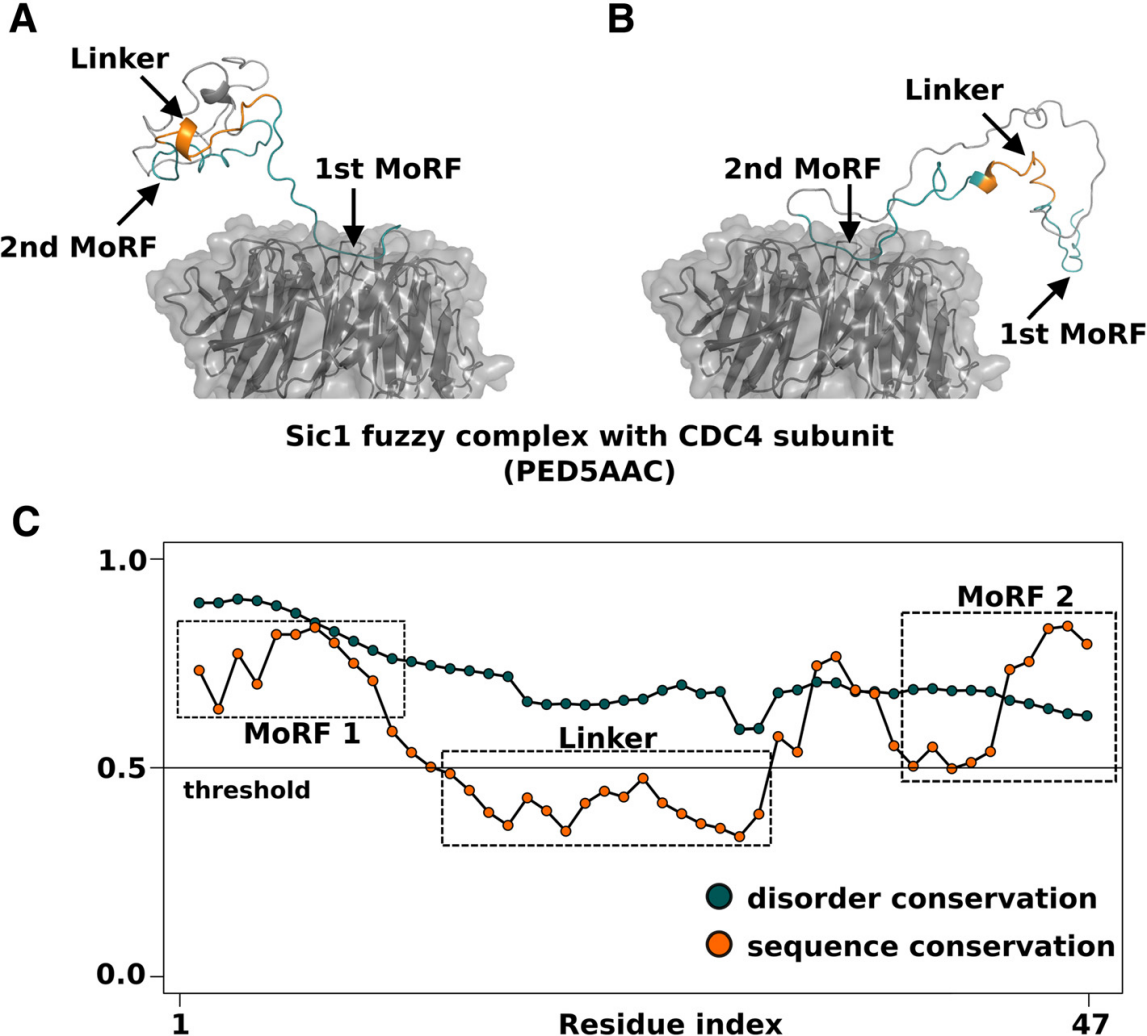
Example of a flexible disordered linker. The intrinsically disordered protein Sic1 binds to the CDC4 subunit of an SCF ubiquitin ligase forming a fuzzy complex (data for the structural ensemble obtained from the Protein Ensemble Database, PED). Two conformations of the same Sic1 fragment from the ensemble of this complex are displayed on panels **A** and **B**. Below, the DisCons profile on panel **C** corresponds to the same fragment, and shows the segment that is flexible disordered between two constrained disordered regions (1st MoRF and 2nd MoRF) that bind to Cdc4. This former segment corresponds to a flexible linker (shown in orange in panels **A** and **B**), which is found between two MoRFs (1st and 2nd on panels **A** and **B**) that bind to the same binding pocket in the fuzzy complex. The flexible conservation of the linker indicates that the sequence is of less importance than its conformational flexibility.

Since ‘flexible’ disordered segments generally function as entropic chains linking structured (or even disordered) segments, finding structural data for them is less straightforward. These residues are often missing from the structures found in the Protein Data Bank (PDB) [18], however, ensemble descriptions of such regions are available from the Protein Ensemble Database (PED) [31]. In order to demonstrate “flexible” disorder, we retrieved the “fuzzy” complex formed between Sic1 and the CDC4 subunit of an SCF ubiquitin ligase [PED:PED5AAC]. As seen generally in “fuzzy” complexes, Sic1 is a fully disordered protein that remains disordered even when bound to its partner. Sic1 has multiple binding segments along its disordered chain that compete for binding to the same pocket on the receptor protein. Figures 5A and B display two conformations (out of the 44 conformers) present in the ensemble description of the complex, while Figure 5C provides the DisCons profile of the corresponding segment of the Sic1 protein. The profile clearly shows that a “flexible” disordered linker connects the two “constrained” disordered binding regions. In several of the 44 different conformations that constitute the ensemble of the “fuzzy” complex, both binding regions are found to contact the binding pocket, whereas the linker segment does not bind to CDC4 in any of the conformations. This indicates that the ‘unstructured-ness’ of the linker is more important for the function of the protein than the corresponding amino acid sequence of this segment.

## Conclusions

DisCons is a novel and freely available online and downloadable tool that combines the quantitative description of the position-specific evolutionary conservation of the amino acid sequence with predictions of the conservation of its disordered/flexible state, providing meaningful information on the evolutionary context of a disordered protein segment. Furthermore, DisCons uses this combined information to classify each disordered position into one of three categories, namely: constrained, flexible and non-conserved. These classes have been suggested to correlate with distinct functions that arise from the disordered state; therefore DisCons may provide information orthogonal to those obtained by other methods, which potentially enhances the reliability of the identification of functionally relevant disordered segments within proteins. We demonstrated that DisCons can be used to investigate both sequence- and disorder conservation in a functionally meaningful manner by bench-marking our procedure on MoRF and SLiM datasets, which are known to be conserved functional units enriched in structural disorder. It is important to emphasize that the success of calculation with DisCons strongly depends on the quality of the underlying multiple sequence alignment; therefore it is advised to review and optimize each MSA to maximize the information of the output. Taken this into consideration, DisCons can be used as an online or stand-alone tool for quantifying the conservation of both sequence and structural disorder by analyzing large-scale protein datasets and individual proteins. As such, DisCons might provide an additional layer of information for the investigation of protein disorder, and could serve to enhance the performance of prediction software such as MoRFPred [13], or provide descriptive information for disorder related databases such as D2P2 [32], MobiDB [33] or PED [31].

## Availability and requirements

DisCons is available as a web application, and as source code, both hosted at http://pedb.vib.be/discons. The source code is written in Python, and has two versions: the multiple sequence alignment (MSA)-based script, and the complete pipeline. The multiple alignment-based version has no requirements (disorder predictor source codes are bundled with the download), while deployment of the full pipeline locally requires the following software: BLAST+ [16] and MAFFT [19]. The software is distributed under the GNU GPL license.

## Abbrevations

IDP: Intrinsically disordered protein
LDR: Long disordered region
MoRF: Molecular recognition feature
MSA: Multiple sequence alignment
SLiM: Short linear motif
